# Letter from a young female physician, *Candidate*

**DOI:** 10.4274/jtgga.2018.0006

**Published:** 2018-06-04

**Authors:** Esra Bilir

**Affiliations:** 1 Koç University School of Medicine, İstanbul, Turkey

To the Editor, 

In 2017, Suzanne Koven, M.D. wrote an outstanding letter to young female physicians (1). In her article, she drew attention to the struggles of the female physicians from different perspectives, as a medical student, a resident, a pregnant woman, an internal medicine specialist, as an academic, but most importantly as a woman! Maybe, she wrote what is already known! Her examples included additional challenges for women, their “pointless” presence at the urology clerkship, being paid less than their male counterparts, “bro humor” in the Department of Obstetrics and Gynecology despite the fact that they were the majority, and higher rates of imposter syndrome being seen in them (1). Nevertheless, a study published by JAMA in 2017 revealed that female internists had better patient outcomes than their male counterparts (2). Another study published by the BMJ in 2017 concluded that female surgeons had lower mortality rates than their male counterparts (3). On the other hand, the percentages of female physicians in the United States of America, Iceland, the European Union, Germany, and Turkey are approximately 34%, 37%, 48%, 46%, and 40% respectively (4,5). Although Turkey is a developing country, it demonstrates less unequal sex distribution among physicians than some of the developed countries (4,5). Hence, I am proud to observe this as a Turkish female medical student. 

As a young female physician, and a very passionate candidate to be a gynecologist, I would like to emphasize that the key message from those two studies is not “women are better internists and surgeons than men” (4,5). As a matter of fact, it is that as physicians, our primary purpose is to exhibit our knowledge and skills for the best interest of our patients for whom we should be ready to accept our limitations and acknowledge our colleagues! Furthermore, above all, we must not discriminate against them based on their sexes but their profession. Hence, we are better together for our patients! 

I read her article on the day that it was published with both delight and sadness. With the above ideas in my mind and my own experience, I wrote a message to her without the hope of that I would receive her reply. Two days later, I was honored to receive her comments regarding my “hopeless” message. First of all, I could not convince myself that as a medical student, I had received an answer from a future colleague from Harvard Medical School and an author of an NEJM article. However, it was real and I started writing a reply with my shaking hands… Since then, Dr. Koven and I have become friends, and I am honored to have her moral support. 

As a medical student, I felt deep sorrow and empathy for Dr. Koven’s experiences. The main reason for it is that in 2017 “we,” the female physicians, are still exposed to a considerable amount of discrimination by our male counterparts and our female counterparts who were trained and worked under harsh conditions. Nevertheless, I must say that I had both the opportunity and the courage to write this letter only thanks to my extraordinary female colleagues namely Dr. Suzanne Koven and Dr. Emine Elif Vatanoğlu-Lutz.
